# Supplementary material for: Silica Nanoparticles Reinforced Ionogel as Nonvolatile and Stretchable Conductors
Source: Membranes (Basel). 2020 Nov 19;10(11):354. doi: 10.3390/membranes10110354 (PMC7699213; doi:10.3390/membranes10110354)
Supplement: Supplementary file 1 [file membranes-10-00354-s001.zip › membranes-971433-supplementary/Supplementary20201116/Supporting Information ionogel-SNP 20201116.pdf]

Electronic Supplementary Information

Silica nanoparticles reinforced ionogel as nonvolatile and stretchable conductors

Shanshan Zhang, Zhen Li, Pengfei Wang*, Pei Huang, Yamei Lu*

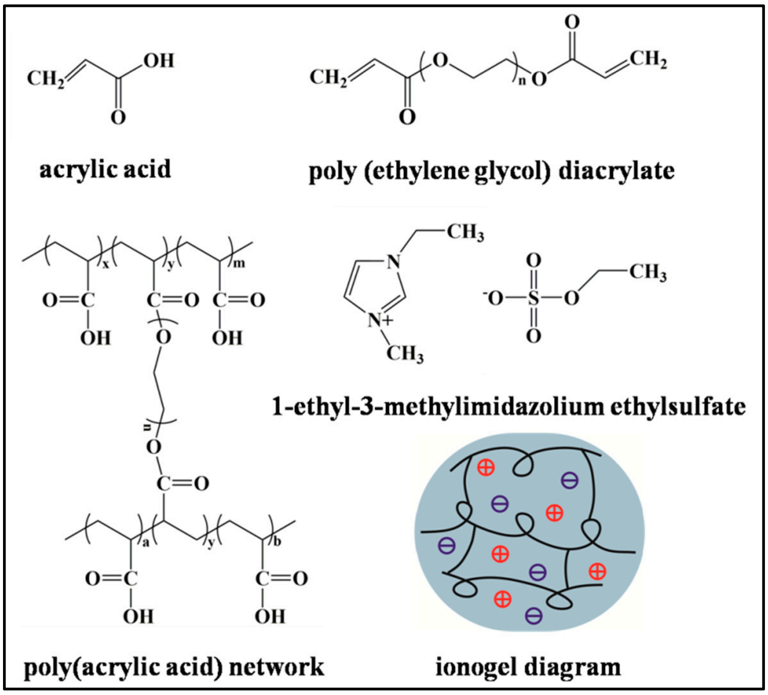

Figure S1. Schematic picture of raw materials and resultant ionogel.

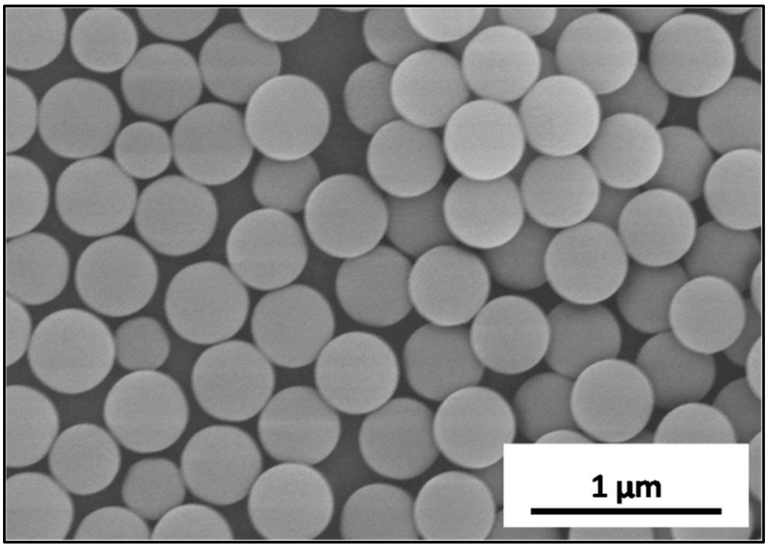

Figure S2. SEM images of Silica nanoparticles (SNP).

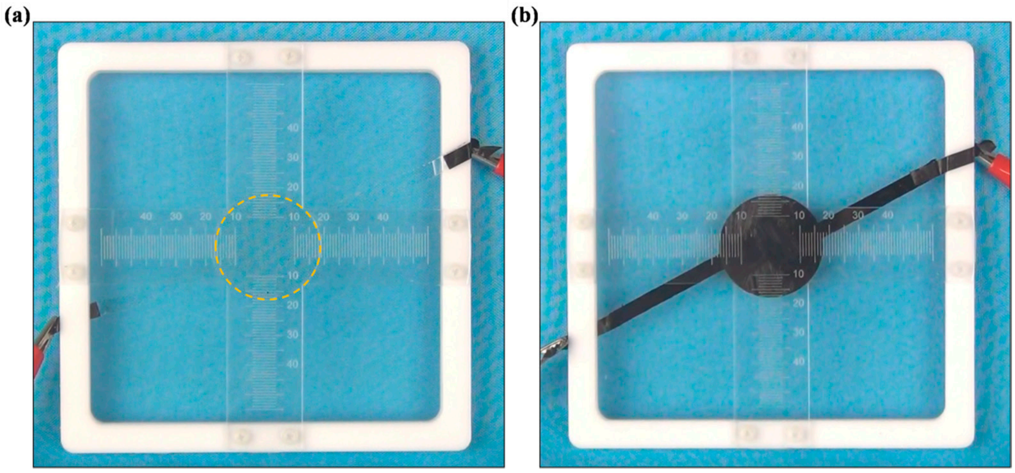

Figure S3. Flexible actuator construction experiment based on: (a) SNP-reinforced ionogel; (b) carbon grease.

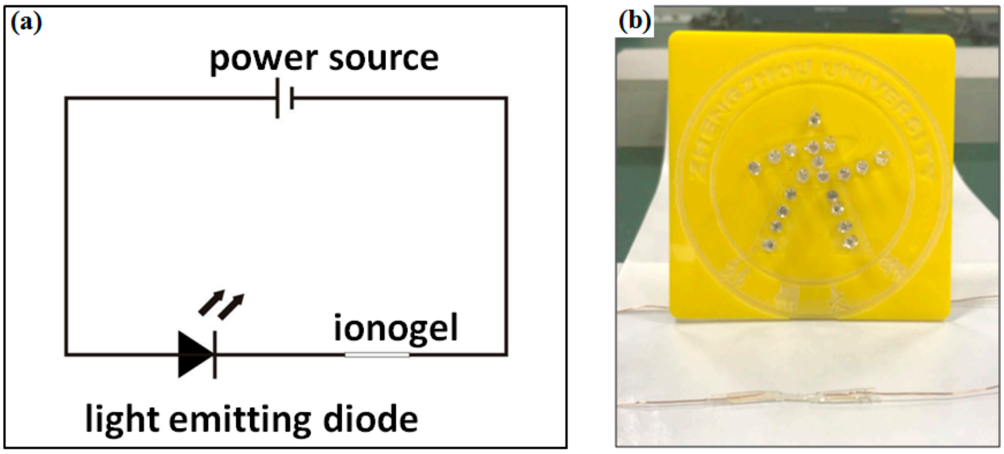

Figure S4: Stretchable cable based on a SNP-reinforced ionogel accessed to the light emitting diode circuit: (a) Circuit diagram; (b) Digital photo.

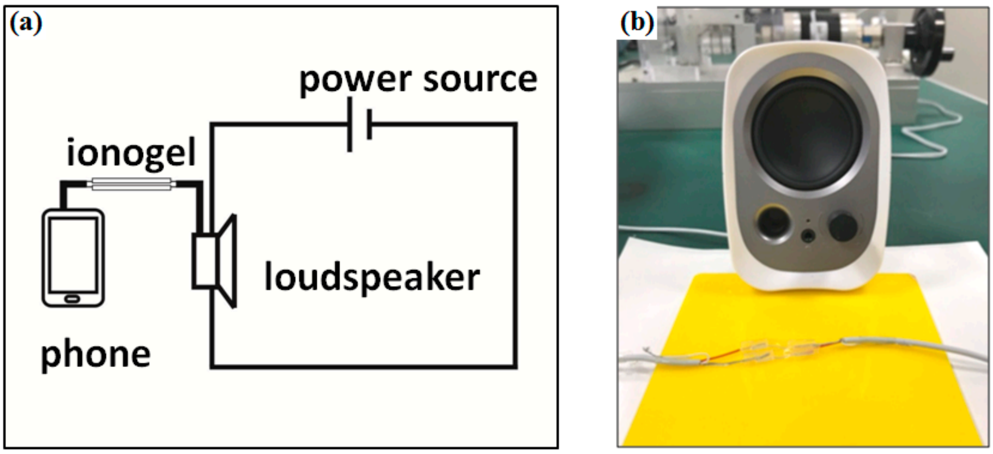

Figure S5. SNP-reinforced ionogel flexible wire accessed to the audio signal line:(a) The schematic diagram; (b) A digital photo.

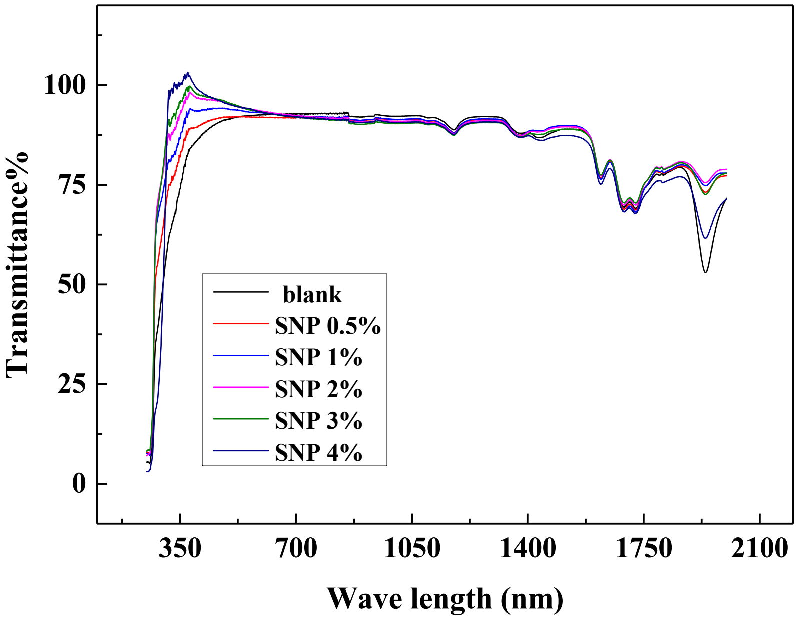

Figure S6. Transmission spectrum images of SNP-reinforced ionogels.

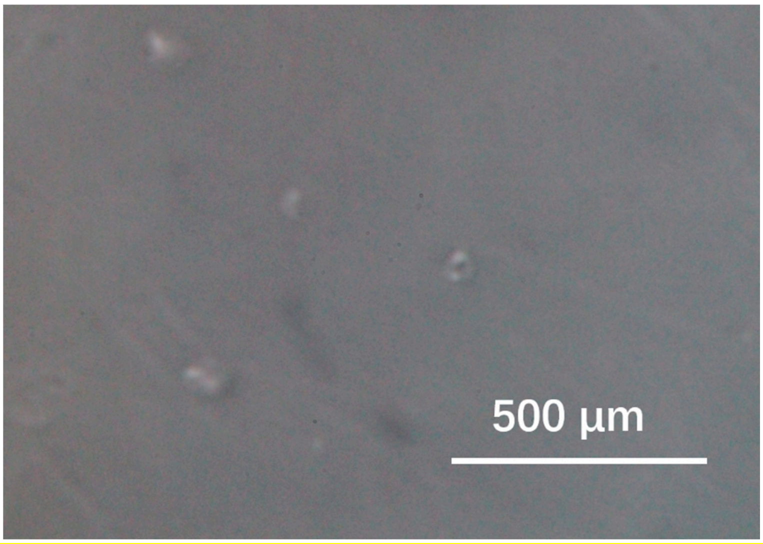

Figure S7. Optical microscope photos for 0.5 wt% SNP-reinforced ionogel.

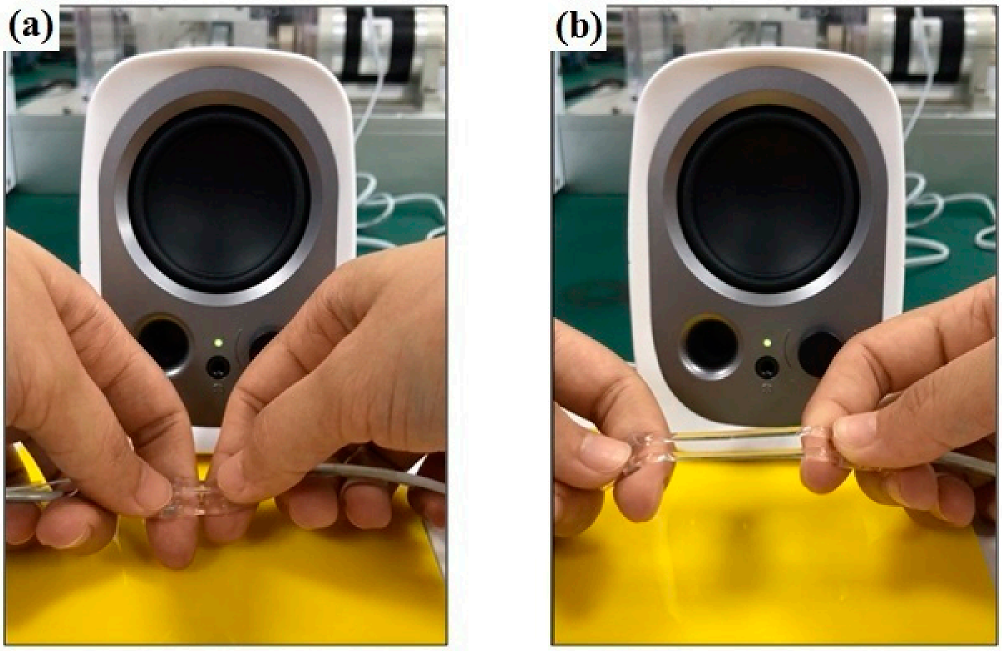

Figure S8. Stretching stretchable cables based on the SNP-reinforced ionogels in audio signal line

(a)A photo of stretchable cables based on SNP-reinforced ionogels without stretching;

(b)A photo of stretching stretchable cables based on SNP-reinforced ionogels.

Table S1 The conductivity of ionogels with different contents of crosslinking agents

Sample	Specific conductance (S/m)
0.8 mol%	1.28 ± 0.07
1.2 mol%	1.26 ± 0.07
1.6 mol%	1.18 ± 0.06
2.0 mol%	1.16 ± 0.06
2.4 mol%	1.23 ± 0.07
