# Supplementary figures and images for: Silica Nanoparticles Reinforced Ionogel as Nonvolatile and Stretchable Conductors
Source: Membranes (Basel). 2020 Nov 19;10(11):354. doi: 10.3390/membranes10110354 (PMC7699213; doi:10.3390/membranes10110354)

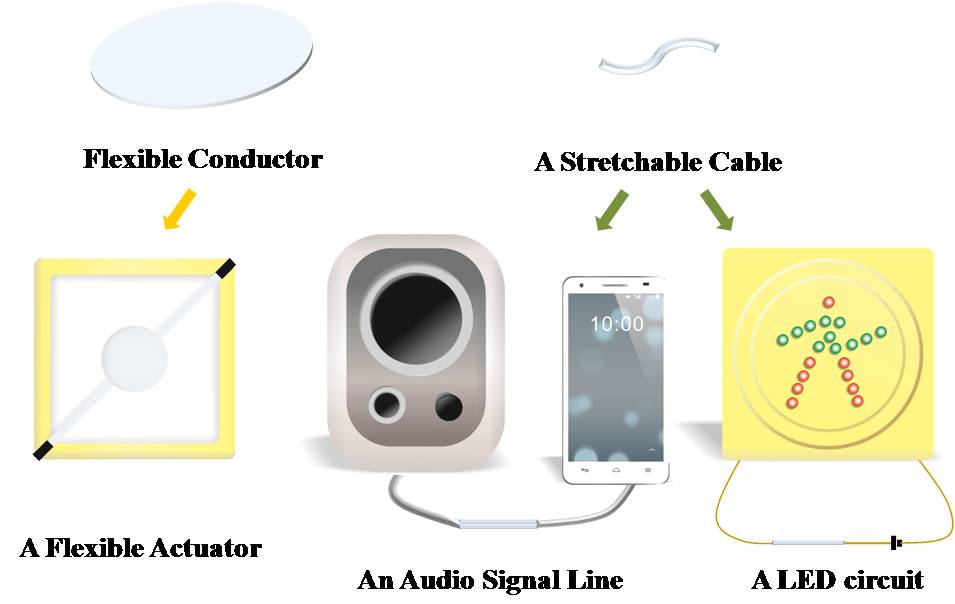

Supplement: Supplementary file 1 [file membranes-10-00354-s001.zip › membranes-971433-supplementary/Supplementary20201116/TOC.jpg]
